# Maximize Lifetime of Wireless Rechargeable Sensor Networks with Mobile Energy-Limited Charging Device

**DOI:** 10.3390/s23187943

**Published:** 2023-09-17

**Authors:** Guoqing Liu, Yaqian Chen, Wanguo Jiao

**Affiliations:** 1 Nanjing Research Institute of Electronics Technology, Nanjing 210039, China; gqliuxd@163.com; 2 College of Information Science and Technology, Nanjing Forestry University, Nanjing 210037, China; chencyq@njfu.edu.cn

**Keywords:** energy management, wireless rechargeable sensor network, charging scheduling strategy, network lifetime

## Abstract

Mobile charging devices (MCDs) have been regarded as a promising way to solve the energy shortage of wireless sensor networks. Due to ignoring some important factors, such as redundant sensor nodes, there is still room to improve network lifetimes. We propose a charging strategy for wireless sensor networks with one energy-limited MCD. To give the best support for sensor nodes which need charging the most, an algorithm is proposed to find the minimum sensor nodes which keep the coverage and connectivity of the network and have the least energy requirements. Then, the goal of maximizing network lifetime is changed into how to utilize the limited energy of the MCD to guarantee the minimum sensor nodes work as long as possible. If the MCD has enough energy for all sensor nodes, the charging algorithm is designed to minimize the outage time of the network and maximize charging efficiency. Otherwise, if the energy capacity is larger than the least energy requirement, the charging target minimizes the outage time of the minimum sensor node; otherwise the charging problem becomes maximizing the lifetime of minimum sensor nodes, which has lower complexity. The results of simulation experiments confirm that our scheme prolongs network lifetime and improves charging efficiency.

## 1. Introduction

Wireless sensor networks (WSNs) are often used to collect basic information and are regarded as sensory organs of the Internet of Things (IoT), which will play an important role of the era of the IoT [1]. With the development of wireless energy transfer technology [2,3], one of the greatest obstacles—energy shortage, which limits the application scale of the WSN—can be overcome. The wireless rechargeable sensor network (WRSN) which employs the mobile charging device (MCD) charging the sensor by using wireless energy transfer technology has attracted many researchers and related industry.

Different from the traditional WSN, which adopts the high energy efficiency algorithm to prolong network lifetime [4,5], works on WRSNs plan the trajectory of the MCD properly to enhance charging efficiency and network lifetime [6,7]. As the sensor node charged by the MCD has the continuous energy source, the WRSN can sustainably work in principle. However, limited by the finite energy capacity and moving speed, some sensor nodes cannot be charged in time because the MCD do not have enough energy to satisfy all charging requirements or cannot reach the sensor node before its energy is exhausted [8].

To address these two problems, many works use multiple MCDs [9,10,11], propose partial charging scheme [12,13,14], or adopt clustering method [15,16,17,18]. In the WRSN using multiple MCDs, MCDs can charge many lifetime-critical sensor nodes simultaneously, which can effectively reduce expired sensor nodes. Though the multiple MCDs method can prolong the network lifetime, to solve the first problem thoroughly it needs a large number of MCDs—the number of MCDs is the same as sensor nodes in extreme situations. Partial charging policy means that, according a certain rule, the MCD leaves a charged sensor node before its battery is full. Therefore, the MCD can replenish energy for more sensor nodes, and the network lifetime can be prolonged. However, this method spends more energy on the movement, which reduces the energy efficiency of the MCD. Through grouping sensor nodes in some clusters, one MCD charges more than one sensor node in the same cluster simultaneously [15,16], or it charges the cluster head which charges the other sensor nodes in the cluster [17,18]. Due to longer energy transfer and secondary energy loss, this method makes the energy more scarce.

These works only study how to efficiently and quickly solve the charging demand of sensor nodes to improve the network lifetime. However, the network lifetime is not only related to the energy supply, but is also related to the energy consumption which determines the distribution of charging requests. Some research in path planning has considered the energy consumption rate of sensor nodes and graded them according to the energy consumption rate of the sensor node and the coverage degree of the target point [18]. However, this research only considers the actual energy consumption without controlling the network energy consumption proactively. To reduce energy consumption, some studies employ the MCD as a mobile data collector [19]. This method reduces the energy consumed by the long distance data transmission which results in larger delay.

In addition to network lifetime, coverage and connectivity are two key and fundamental metrics which evaluate the functionality performance of the WSN. There is little research considering both network connectivity and target coverage in the charging scheduling of the WRSN. Almost all existing studies assume that all sensor nodes work simultaneously, and the network lifetime is defined as the time the WSN has worked when one or a certain proportion of sensor nodes die. However, after one sensor node dies, all targets may be still covered and collected data can be transmitted to Sink. That is, the WSN can still work, and the actual network lifetime is longer. Meanwhile, when one sensor node dies, the target cannot be covered or the network is not connected, and then the proportion definition is incorrect. Therefore, the definition of the network lifetime should consider the connectivity and coverage, and the end time of the WSN in our work is defined as the moment that the network is not connected or one target cannot be covered. Thus, the role that the sensor node plays in the coverage and connectivity should be considered in charging scheduling.

To overcome the above shortages, in this work we propose a charging algorithm to optimize the charging efficiency and network lifetime which considers the connectivity and coverage. Firstly, according to the position and energy consumption rate of the sensor node, a set of sensor nodes with smallest energy consumption which satisfies the coverage and connectivity requirement is found. That is, the minimum energy demand to keep the network working is obtained. Then, according to the relation between the energy requirement and the energy capacity of the MCD, we design three optimal charging algorithms to maximize network lifetime and charging efficiency.

The contribution of this work can be concluded as follows. First, we analyze the relation between the energy consumption rate and the network parameter, and then an scheme maximizing network lifetime is proposed considering both coverage and connectivity. Second, according to the supply–demand relationship, we design a charging algorithm with different targets. Our work firstly combines energy management and charging scheduling to optimize energy efficiency and charging efficiency simultaneously in the WRSN.

The remainder is organized as follows. Section 2 will introduce the related work. Then, Section 3 describes the network model, while the problem is analyzed in Section 4. The proposed framework is explained in Section 5. We evaluate and demonstrate the performance of the proposed method in Section 6. At last, the whole paper is summarized in Section 7.

## 2. Related Work

The main problem of the WRSN is how schedule to the MCD to charge sensor nodes in time. In order to solve multiple simultaneous charging requirements and limited capacity of the MCD, here we introduce three kinds of works: multiple MCDs [9,10,11], partial charging scheme [12,13,14], and clustering method [15,16,17,18].

When the WRSN has multiple MCDs, many lifetime-critical sensor nodes can be charged simultaneously, and expired sensor nodes can be effectively reduced. In [9], an approximation algorithm was proposed to minimize the longest charging delay through finding the closed charging tour for each MCD. As the battery capacity of different MCDs may be different, the charging scheme considering the battery capacity of the MCDs was proposed to plan charging tours and depot positions of MCDs in [10]. Authors in [11] proposed an online charging architecture which makes MCDs collaborate dynamically to minimize the energy hole rate. These works indicate that more sensors are charged in time and inevitably die in the WRSN with multiple MCDs. Thus, using multiple MCDs can prolong network lifetime, especially in the larger scale WSN. However, there are still some open problems, such as the optimal number of MCDs, which should consider the tradeoff among the utilization of the MCD, device cost, and the gain of the network lifetime.

In partial charging policy, as the sensor node is not fully charged, more sensor nodes can get chances to be charged, and then dead sensor nodes will be reduced accordingly. In [12], a partial charging scheme was proposed to minimize the overall dead time of sensor nodes through selecting core sensor nodes based on the contribution of sensor nodes on charging performance. Different from [12], authors in [13] minimized not only the overall dead time but also the number of dead sensor nodes. Similarly, the mechanism in [14] minimizes dead sensor nodes and maximizes energy utilization. These works show that dead sensor nodes are indeed reduced, even up to 70%. Meanwhile, the moving distance of the MCD is longer, and more energy is spent on extra moving. Thus, improving charging efficiency is the main challenge faced by these works.

Clustering lets one MCD charge more than one sensor node simultaneously. There are two methods: multiple charging method [15,16] and closed node charging method [17,18]. Multiple charging method can improve charging efficiency, but the charging delay of a cluster is determined by the longest charging time of the sensor node in the cluster, which will result in time waste, especially in the uneven network. To address this problem, partial charging is combined with the clustering method [14]. Closed node charging method can reduce charge anchors, and then allows the MCD to serve more sensor nodes. However, as the charging efficiency between the cluster head and the sensor node cannot reach 100%, the cluster head should also have charging ability. Hence, its charging efficiency is not high and the hardware requirement of sensor nodes is high.

The above-mentioned three types of works focus on how to schedule the MCD to charge, ignoring the energy consumption. If the energy consumption is reduced, given the same energy supply, more sensor nodes can be charged and the network lifetime can be prolonged. To reduce energy consumption, some works employ the MCD as a mobile data collector [19]. This method reduces the energy consumed by the long distance data transmission which results in a larger delay. To address this, a mobile base station is used to relay collected data while the MCD is responsible for charging the sensor nodes in time; for example, in [20], the author designed an on-demand charging architecture to maximize the survival rate of sensor nodes and coverage of targets.

Despite the above-mentioned works about two-dimensional WRSNs, little research into the three-dimensional WRSN with certain applications has been proposed recently. However, in [21], the author proposed a charging scheme to schedule unmanned aerial vehicles to charge sensor nodes on bridges.

Before introducing our work, Table 1 for the main used symbols is given.

## 3. The System Model

**Figure 1:**
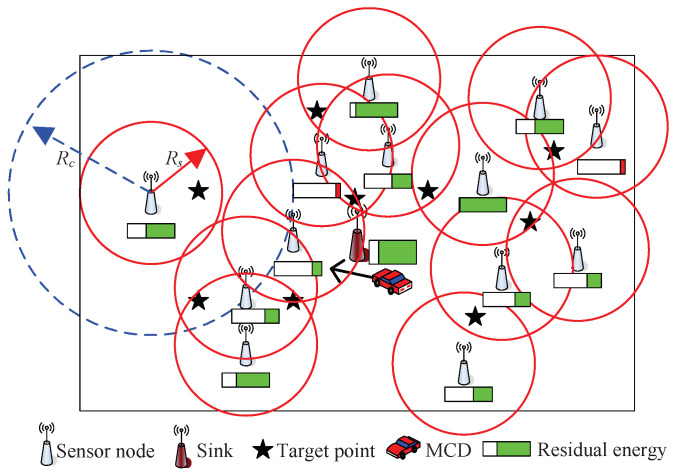
Network scenario.

The considered network scenario is shown in Figure 1, which includes an MCD, many sensor nodes, and some monitored target points. The energy management and charging scheduling involves network parameters, energy consumption, and the charging method. Based on Figure 1, we introduce the network model, charging model, and energy model in the following.

### 3.1. Network Model

Suppose that the monitoring area is a two-dimensional rectangle plane *A*, and the area of *A* is $L_{1}\times L_{2}$. There are *N* sensor nodes randomly placed on the plane *A*, which is widely adopted in the WSN, such as [22,23]. The set of sensor nodes is $S=\left\{s_{1},s_{2},\ldots ,s_{N}\right\}$. The coordinate of each sensor node is known, and the coordinate of $s_{i}$ is denoted by $\left(x_{i},y_{i}\right),1\leq i\leq N$. The *M* target points are randomly distributed in area *A* with fixed positions and the coordinate of the target point is known. The set of target points is $P=\left\{p_{1},p_{2},\ldots p_{M}\right\}$. The coordinate of target point $p_{j}$ is $\left(x_{j},y_{j}\right),1\leq j\leq M$. We assume that the sensor network is static and cannot move. To guarantee all target points are covered, *N* and *M* should satisfy N≫M. The network lifetime $N_{L}$ is defined as the time that the network has worked when a target is not covered or the network is not connected.

The sensing radius is $R_{s}$. A binary variable $B_{ij}$ is used to indicate whether sensor node $s_{i}$ can cover target point $p_{j}$, which is defined as:

(1) $$B_{ij}=\left\{\begin{array}{c} 1,\;\mathrm{if}\;d_{ij}\leq R_{S}\\ 0,\;\mathrm{if}\;d_{ij}>R_{S} \end{array}\right.,$$

where $d_{ij}$ is the distance between target point $p_{j}$ and sensor node $s_{i}$. As we assume that the network is a two-dimensional plane, the distance between two nodes is Euclidean distance which is often used by related works [24] and can be calculated as $d_{ij}=\sqrt{{\left(x_{i}-x_{j}\right)}^{2}+{\left(y_{i}-y_{j}\right)}^{2}}$. If the distance is bigger than $R_{s}$, $B_{ij}=0$, which indicates that target point $p_{j}$ cannot be covered by sensor node $s_{i}$. Otherwise, $B_{ij}=1$, and the sensor node can monitor the target node.

The communication radius of each sensor node is $R_{c}$. In order to ensure all collected data be sent to Sink, the network must be fully connected. According to the proof in [25], when the communication radius and the sensing radius satisfy $R_{c}\geq 2R_{s}$, the network is fully connected if all targets are covered. In this paper, we assume that $R_{c}\geq 2R_{s}$. Thus, only if all targets are covered, the requirement of the network connectivity is guaranteed. Thus, the network lifetime is revoiced as the time which the network has worked at the moment the target is not fully covered. Moreover, the ideal channel model is assumed to be used. That is, all channels among all sensor nodes and Sink are reliable and error-free. There is no transmission failure due to bit error.

The data generation rate of sensor node $s_{i}$ is denoted by $\lambda_{i}$, and the relayed date rate from sensor node $s_{i}$ to sensor node $s_{j}$ is $f_{ij}$. The neighbor set of sensor node $s_{i}$ is $N_{i,ei}$, thus the total data $\lambda_{i,total}$ transmitted by sensor node $s_{i}$ can be expressed as:

(2) $$\lambda_{i,total}=\lambda_{i}+\sum_{s_{j}\in N_{i,ei}}f_{ji}.$$

### 3.2. Recharging Model

The initial energy $E_{0}$ of the sensor node is full, that is $E_{0}=E_{\max}$. Assume that the minimum energy required to maintain the basic function of sensor node is $E_{\min}$. When the remaining energy is smaller than $E_{\min}$, the sensor stops working and becomes a sleep node. If the residual energy is less than a certain value, which is denoted by $E_{th}$, the sensor node needs to be charged and sends a charging request. For example, $E_{th}=0.6E_{\max}$. Further, it is assumed that Sink knows the position and energy information of the sensor node. Sink sends this collected information to the MCD and the MCD arranges the travel path according to a certain rule.

In the network, we assume that one MCD is responsible for energy replenishment of all sensor nodes and the energy capacity of the MCD is $B_{\max}$ which is limited. The MCD starts from Sink, charges demanding sensor nodes one by one, and returns to Sink after finishing the charging task or when its energy can only afford its way back. Thus, Sink is not only the data collector and network controller, but also the energy supplier of the MCD. The running path of the MCD will form a closed-loop path, which can be expressed by $P_{ath}=\left\{s_{0}^{\prime },s_{1}^{\prime },\cdots ,s_{L-1}^{\prime },s_{L}^{\prime }\right\}$. As the path starts from Sink and ends at Sink, both $s_{0}^{\prime }$ and $s_{L}^{\prime }$ are Sink. That is, there are (L−1) sensor nodes on the charging path $P_{ath}$ and $s_{i}^{\prime }$ is the *i*-th charged sensor node, which may not be $s_{i}$. The moving speed of the MCD is constant which is denoted by $V_{M}$.

When the MCD charges a sensor node, it will stop charging until the sensor node is fully charged. Hence, the required energy of the sensor node $s_{i}$ is:

(3) $$E_{i,r}=E_{\max}-E_{i,t},$$

where $E_{i,t}$ is the residual energy of sensor node $s_{i}$ at the moment *t* that it is charged. If sensor node $s_{j}$ does not send the charging request, the required energy $E_{j,r}=0$.

Meanwhile, the charging time $T_{i}$ of sensor node $s_{i}$ can be calculated as:

(4) $$T_{i}=\frac{E_{i,r}}{P_{i,in}},$$

where $P_{i,in}$ is the charging rate of sensor node $s_{i}$.

The cycle time of the MCD consists of two parts: the charging time $T_{ch}$ of the MCD to the sensor node and the moving time $T_{tr}$ spent by the MCD from one sensor node to the next one. Thus, the cycle time can be expressed as:

(5) $$T_{cyc}=T_{ch}+T_{tr}=\sum_{i=1}^{L-1}T_{i,r}+\sum_{l=0}^{L-1}\frac{d_{l,l+1}}{V_{M}},$$

where $T_{i,r}$ is the charging time of sensor node $s_{i}^{\prime }$ on the path, which can be obtained by using $T_{i}$.

According to the collected information, the MCD determines sensor nodes to be charged and the charging order.

### 3.3. Energy Model

The energy consumption in the network mainly consists of two parts. One part is consumed by the sensor node, and the other part is consumption of the MCD. The energy consumption of sensor nodes is further divided into three main portions: the energy used to sense data, sending data, and receiving data. Let $e_{s}$ denote the energy consumed by sensing one bit of data, then the energy of sensor node $s_{i}$ used to sense data is:

(6) $$C_{i,S}=e_{s}\lambda_{i}.$$

The energy consumed by receiving data from neighbors is expressed as:

(7) $$C_{i,R}=\sum_{s_{j}\in N_{i,ei}}e_{r}\left(d_{ji}\right)f_{ji},$$

where $e_{r}\left(d_{ji}\right)$ is the energy consumption of receiving the unit data from sensor node $s_{j}$ to sensor node $s_{i}$.

Since the data transmission adopts the multi-hop manner, the energy consumed by the data transmission including its own generated data and the relayed data from adjacent sensor nodes is expressed as:

(8) $$C_{i,T}=\sum_{s_{j}\in N_{i,ei}}e_{t}\left(d_{ij}\right)f_{ij}+e_{t}\left(d_{i(nh)}\right)\lambda_{i}$$

where $e_{t}\left(d\right)$ is the energy consumption of transmitting the unit data when the distance between the received sensor node and the transmit sensor node is *d*, $d_{i(nh)}$ is the distance between sensor node $s_{i}$ and the next-hop sensor node.

Thus, by combining Equations (6)–(8), the energy consumption rate of sensor node $s_{i}$, denoted by $C_{i}$, can be calculated as:

(9) $$C_{i}=\left(e_{s}+e_{t}\left(d_{i(nh)}\right)\right)\lambda_{i}+\;\;\sum_{s_{j}\in N_{i,ei}}e_{r}\left(d_{ji}\right)f_{ji}+\;\;\sum_{s_{j}\in N_{i,ei}}e_{t}\left(d_{ij}\right)f_{ij}$$

Notation $P_{out}$ indicates the charging rate of the MCD to the sensor node. According to [26], the relationship between $P_{out}$ and $P_{i,in}$ is expressed as:

(10) $$P_{i,in}=P_{\mathrm{out}\;}\times \mu \left(d_{i,M}\right),$$

where $\mu (d_{i,M})$ is a function of the distance between the MCD and the charged sensor node and $\mu \left(d_{i,M}\right)\in \left[0,1\right]$. In this work, the MCD can be closed to the sensor node when it charges this sensor node within a smaller distance. That is, the near-field charging is adopted. Thus, we assume that the MCD has a fixed energy loss when it charges the sensor nodes [3], such as μ(d)=0.5.

Substituting Equation (10) into Equation (4), the energy of the MCD used to charge sensor node $s_{i}$ is obtained as $E_{i,r}/\mu \left(d_{i,M}\right)$. Then, the energy of the MCD used to charge sensor nodes is expressed as:

(11) $$E_{ER}=\sum_{s_{i}\in P_{ath}}\frac{E_{i,r}}{\mu (d_{i,M})}.$$

Let $e_{0}$ denote the energy consumption of the MCD moving one meter and the energy spent on the moving trajectory is $e_{0}V_{M}T_{tr}$. By using Equations (5) and (11), the energy required by the MCD in a round is calculated as:

(12) $$E_{r,MCD}=\sum_{s_{i}\in P_{ath}}\frac{E_{i,r}}{\mu (d_{i,M})}+\sum_{l=0}^{L-1}e_{0}d_{l,l+1}.$$

## 4. The Problem Formulation and Analysis

As the energy capacity and moving speed of the MCD are limited, the sensor node may not get a charging chance or be charged in time, and then the network cannot unceasingly work due to energy exhaustion of the sensor node. Maximizing network lifetime is a primary target of the WRSN, which is also the object of this work. Based on the above description, the considered problem can be described as:

(13)maxNL(13a)s.t.Er,MCD<Bmax,(13b)Ci<Ei,t,i=1,2,⋯,N(13c)∑i=1NBij≥1,j=1,2,⋯,M(13d)crad(Ni,ei)≥1,i=1,2,⋯,N

where $\mathrm{crad}(N_{i,ei})$ is a function used to get the number of elements in set $N_{i,ei}$.

According to the problem described by Equation (13), to guarantee the network lifetime, the MCD should charge all sensor nodes which send charging requests in time. However, due to limited energy capacity, the MCD may not satisfy all charging requests. In practice, many redundant sensor nodes are used to enhance the coverage and connectivity quality, and the data transmission of these sensor nodes will consume larger amounts of energy. If these sensor nodes go to sleep, the amount of transmitted data is reduced and energy consumption of the sensor node will be reduced. Then, the energy requests are decreased accordingly, and the energy of the MCD may be enough for the network. Meanwhile, the sensor nodes which urgently need energy have the chance to be charged in time. Hence, finding out the set with minimum sensor nodes is important to maximize network lifetime.

As the number of sensor nodes is much larger than targets points, it is easy to validate that there must be at least one set which has the minimum sensor nodes to cover all target points and guarantee the connectivity. Suppose that the number of nodes-minimum sets is *q* and q≥1. The set of the nodes-minimum sets is denoted by $Q=\left\{V_{\mathrm{mincov}}^{1},\cdots ,V_{\mathrm{mincov}}^{q}\right\}$, where $V_{\mathrm{mincov}}^{i}$ is a nodes-minimum set which is expressed as $V_{\mathrm{mincov}}^{i}=\left\{s_{1},\cdots ,s_{v_{i}}\right\},v_{i}\leq N$.

Though the nodes-minimum set has minimum sensor nodes, it ignores energy consumption rate and residual energy of the sensor node. Thus, it cannot minimize the energy consumption and the required energy of keeping the network. Based on nodes-minimum sets, the set which has the minimum energy request is further searched due to the following theorem.

**Theorem** **1.** *The network lifetime is maximized if the lifetime of the set with the minimum energy requirement is maximized.*

**Proof.**  The set which has minimum energy request is denoted by $V_{\mathrm{minreq}}$ and the minimum required energy is $E_{mr}$. According to the relation among the required energy $E_{r,MCD}$, $E_{mr}$, and $B_{\max}$, there are three cases: Case 1, $E_{mr}>B_{\max}$, Case 2, $E_{r,MCD}>B_{\max}>E_{mr}$, and Case 3, $B_{\max}>E_{r,MCD}$.    □

For Case 1, the energy of the MCD cannot satisfy the minimum requirement. The optimal charging path of the MCD and the corresponding network lifetime are denoted as $P_{ath}^{*}$ and $N_{L}^{*}$, respectively. There is another charging scheduling policy whose charging path is $P_{ath}^{**}$ and the achieved network lifetime is $N_{L}^{**}$. Suppose that $N_{L}^{**}>N_{L}^{*}$.

As the nodes-minimum set limits the total amount of working sensor nodes and the generated data, the energy consumption rate of the nodes-minimum network is smaller. That is, the network should consume more energy to support the same lifetime than the nodes-minimum network, much less $N_{L}^{**}>N_{L}^{*}$. Thus, the required charging energy $E_{R}^{*}$ and $E_{R}^{**}$ of the nodes-minimum network and the network satisfy $\left|E_{R}^{*}\right|>\left|E_{R}^{**}\right|$.

The set with the minimum energy requirement is an element of the set Q, and thus has the minimum sensor nodes. That is, $\left|P_{ath}^{*}\right|<\left|P_{ath}^{**}\right|$ where $\left|P_{ath}^{*}\right|$ is the number of the sensor nodes on the charging $P_{ath}^{*}$. To traverse all sensor nodes on the path, the moving distance of the MCD on the $P_{ath}^{**}$ is larger.

According to Equation (12), the MCD will consume more energy on the $P_{ath}^{**}$. However, in this case, the total energy which can be used is the same as the energy capacity of the MCD. Therefore, the hypothesis does not hold and the network has the same lifetime as $N_{L}^{*}$.

For Case 2, the MCD has enough energy to charge all sensor nodes in the nodes-minimum set, and there must be a charging algorithm to guarantee the sensor nodes be charged. Then, the network will sustainably work, and the network lifetime is infinite. That is, the network lifetime is maximized.

For Case 3, the energy of the MCD is sufficient for all sensor nodes. Similar to Case 2, both the minimum set and the network have the same lifetime.

Therefore, the maximum lifetime of the network can be obtained through maximizing the lifetime of the network constructed by the set with the minimum energy requirement.

**Corollary** **1.** *If the minimum energy requirement is smaller than the energy capacity of the MCD, the network lifetime maximization is changed into minimizing the sleep time of sensor nodes in **V**_minreq_*.

Therefore, the problem in Equation (13) can be solved in two stages. Firstly, find out the energy-minimum set based on the nodes-minimum sets and the minimum energy requirement. Then, according to the relation between the minimum required energy and the energy capacity of the MCD, the different charging scheduling algorithms are designed to achieve network lifetime optimization.

## 5. Proposed Framework

As mentioned in the previous section, in order to maximize the network lifetime, we need to first find out the minimum energy requirement set $V_{\mathrm{minreq}}$ and the minimum required energy $E_{mr}$. According to Theorem 1, the charging algorithm should be designed separately based on the relation among $E_{mr}$, $B_{\max}$, and $E_{r,MCD}$.

In Case 1 ($E_{mr}>B_{\max}$), the energy of the MCD is insufficient to fully charge all sensor nodes within $V_{\mathrm{minreq}}$. To maximize the lifetime, a partial charging strategy is proposed to determine the charging order and the energy to be replenished for each senor node.

In Case 2 ($E_{r,MCD}>B_{\max}>E_{mr}$), the MCD has enough energy to replenish the sensor node in the set $V_{\mathrm{minreq}}$, but not the whole network. As discussed before, the network can sustainably work since sensor nodes guaranteeing the coverage and connectivity can get enough energy supply. According to Corollary 1, the main target of the charging policy is minimizing the sleep time of the sensor node in the set $V_{\mathrm{minreq}}$. To enhance charging efficiency and reduce further charging requests, a scheduling algorithm should be designed to charge sensor nodes out of the set.

In Case 3 ($E_{r,MCD}<B_{\max}$), the energy of the MCD can satisfy charging requests of all sensor nodes. Hence, to guarantee the coverage and connectivity, the MCD charges sensor nodes in the set $V_{\mathrm{minreq}}$ with the target of minimizing the sleep time. Then, the sensor node out of the set is charged with the target of maximizing charging efficiency.

**Figure 2:**
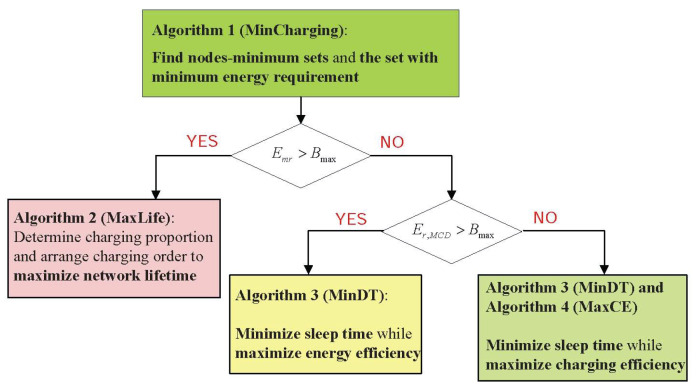
The proposed framework.

Therefore, to maximize network lifetime and fully utilize the energy of the MCD, a scheduling and charging framework is proposed, which is shown in Figure 2. First, an algorithm is proposed to find the set which has the minimum sensor nodes and least required energy. Then, according to the energy relation, algorithms with different targets are proposed.

### 5.1. Find Minimum Energy Requirement

As shown in Figure 2, the first step of the framework is determining the set which has the minimum energy demand. Through analyzing $E_{r,MCD}$, it can be found that the traveling distance of the MCD and the energy requirement of sensor nodes are two the main factors [27]. To minimize the energy consumption, the active sensor nodes should be as few as possible while the traveling distance of the MCD is as short as possible. To guarantee the application, all target points should be covered and all active sensor nodes must connect with each other. Finding a set satisfying all above demands is difficult.

To solve this difficulty, we divide the solution into two stages. First, find the minimum node set which covers all target points [28]. As there may be more than one such set, then find the set with the minimum energy demand from the set of minimum-nodes sets. Since the coordinates of nodes are known and the number of nodes is limited, we can calculate the minimum energy demand $E_{mr}$ by using shortest path routing and find out the node set $V_{\mathrm{minreq}}$ with minimum energy demand.

**Figure 3:**
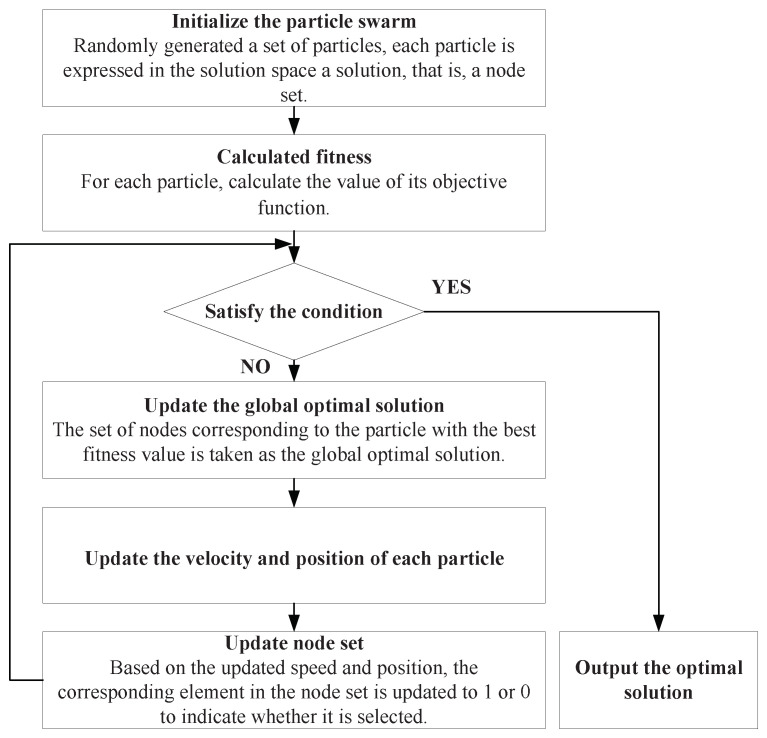
Flowchart of Particle Swarm Optimization Algorithm.

Searching minimum node sets has proven to be an NP-hard problem; thus, we proposed a MinCharging algorithm based on particle swarm operation algorithm to find the minimum node set $V_{\mathrm{minreq}}$ and calculate minimum energy demand $E_{mr}$. In the particle swarm optimization algorithm, particles can be defined as individuals in a population that represent potential solutions to a given problem. Each particle has a position and velocity in the search space, and through continuous iterations, it aims to converge to the optimal solution. In this paper, each particle is defined as a set of charging nodes that minimize the required energy. The flow chart of applying particle swarm optimization to solve the minimum node set is shown in Figure 3.

The designed algorithm uses binary numbers 0 and 1 to represent whether the sensor node is included in the set $V_{\mathrm{mincov}}^{i}$, where 1 and 0 represent whether or not the corresponding sensor node is in the set, respectively. To satisfy the requirement of the minimum node set, the fitness function is proposed by considering two following objectives.

Objective 1, all target points should be covered and the coverage rate is as large as possible.

(14) $$fit_{1}=\max\theta^{k}=\frac{{∥n\left(k\right)∥}^{2}}{M},$$

where ${∥n\left(k\right)∥}^{2}$ represents the number of target points which can be covered by sensor nodes on the *k*th particle.

Objective 2, the number of selected sensor nodes should be the least. In order to maintain consistency, we convert the target to the maximum number of unselected sensor nodes to facilitate the calculation of our minimum energy requirement sensor node set.

(15) $$fit_{2}=\max\sigma =1-\frac{\sum_{i=1}^{n}s_{i}}{N},$$

where $\sum_{i=1}^{n}s_{i}$ represents the number of selected nodes.

The function of *k* chromosome is denoted by fitness(k) which is defined as:

(16) $$fitness\left(k\right)=\alpha fit_{1}+\left(1-\alpha \right)fit_{2}-punish,$$

where 0<α<1. It can be noticed that the larger the value of the objective function, the higher the superiority. Meanwhile, we apply a penalty term in the fitness function to penalize particles that do not satisfy the requirements. Specifically, when a particle violates the requirements, such as having charging nodes that are not required, we randomly assign a large positive value to decrease the fitness value of that particle, thus penalizing it. If a particle satisfies all the requirements, the penalty term is set to 0. Through maximizing the value of fitness function, the set $V_{\mathrm{mincov}}^{i}$ can be found.

Then, based on the set Q, calculating the minimum energy requirement of each set $V_{\mathrm{mincov}}^{i}$. Through comparing the value of the minimum energy requirement, the set $V_{\mathrm{minreq}}$ with minimum energy demand node set and minimum energy demand $E_{mr}$ are obtained. More details can be found in Algorithm 1.

**Figure 4:**
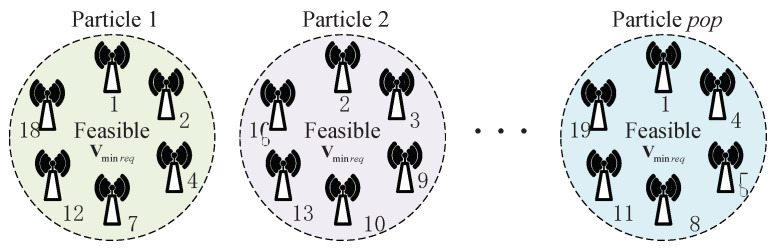
The legend for particles.

To illustrate the finding process, the legend for particles is given, which is shown in Figure 4 where pop represents the number of particles.
**Algorithm 1** Charging Minimum Energy Demand Algorithm1:**Input**:$\left(x_{j},y_{j}\right)$, $R_{S}$, $\left(x_{i},y_{i}\right)$, $E_{\max}$, $V_{M}$, $B_{\max}$, pop, and iter.2:**Output**: Minimum energy demand $E_{mr}$ and minimum energy demand node set $V_{\mathrm{minreq}}$.3:Initializes the selected value $n_{i}$ for each node;4:For i=1 : *N*5:if $n_{i}>\xi$, $n_{i}=1$, endif.6:For j=1:pop7:Calculate the fitness fitness according to Equation (16);8:Select the globally optimal particle and the corresponding fitness function value;9:For k=1:iter10:For l=1:pop11:Update particle velocity;12:Update particle position;13:Calculate the fitness fitness;14:Select the globally optimal particle and the corresponding fitness function value;15:Update the minimum cover node set $V_{\mathrm{mincov}}$;16:Nodes in the set are sorted according to the distance from MCD, and $V_{\mathrm{mincov}}$ is updated;17:Calculate the energy required by the sensor nodes in $V_{\mathrm{mincov}}$ according to Equation (12) and the shortest travel distance of corresponding nodes, and derive $E_{req}$;18:End all for circulations19:Repeat above operations. The energy required for charging the sensor nodes in the set under different $V_{\mathrm{mincov}}$ is obtained. $E_{mr}$ and $V_{\mathrm{minreq}}$ at this time are obtained by ordering $E_{req}$.

### 5.2. The Algorithm Maximizing Network Lifetime

Based on the previous subsection, we have obtained the minimum energy demand $E_{mr}$ and sensor nodes to be charged. If $E_{mr}$ is larger than the energy capacity of the MCD, the energy of MCD is insufficient to serve all sensor nodes within $V_{\mathrm{minreq}}$, no more than the whole network, so a partial charging strategy is adopted for sensor nodes within the set to maximize the network lifetime.

According to the definition of the set $V_{\mathrm{minreq}}$, the network lifetime in this case means the time which the network has work when one sensor node in the set $V_{\mathrm{minreq}}$ is dead. Hence, if each sensor node has the same work time after being charged by the MCD, the network lifetime achieves the maximum value. Considering the residual energy and the energy consumption rate of the sensor node, the partial charging parameter $\delta_{i}$ is expressed as:

(17) $$\delta_{i}=\frac{E_{i,t}+E_{i,charge}}{C_{i}},$$

where $E_{i,charge}$ is the energy used to replenish sensor node $s_{i}$.

According to Equation (17), the MCD calculates the energy used to replenish sensor node $s_{i}$. Then, a shortest-path algorithm is used to determine the charging path to minimize the energy consumed on the moving path. The specific charging policy is given in Algorithm 2.
**Algorithm 2** Algorithms to maximize network lifetime1:**Input**: $V_{\mathrm{minreq}}$ and $E_{mr}$.2:**Output**: Charging node sequence and $E_{i,charge}$.3:Initialize the charging set $V_{charge}=V_{\mathrm{minreq}}$;4:For $i=1:\left|V_{\mathrm{minreq}}\right|$5:Calculate the remaining working time of each sensor node Ti,rest=Ei,rEi,rCiCi;6:Calculate the energy to be charged $E_{i,charge}$ of the sensor nodes in the $V_{\mathrm{minreq}}$ according to Equation (17);7:End for.8:Sensor nodes in $V_{\mathrm{minreq}}$ are sorted in ascending order of distance from $s_{i}$;9:Update the charging set $V_{\mathrm{charge}}$;10:For $i=1:\left|V_{\mathrm{charge}}\right|$11:Calculate the energy $E_{i,charge}$ of each sensor node to be charged;12:End for.13:Derive charging path according to $E_{i,charge}$ and updata the energy of each sensor node to be charged.

### 5.3. The Algorithm Minimizing the Dead Time

If $E_{mr}<B_{\max}$, the MCD has additional energy after charging all sensor nodes in the set $V_{\mathrm{minreq}}$. Although the MCD has enough energy for the sensor node in the set, due to limited moving speed, some sensor nodes may not be charged in time. Minimizing the sleep time of sensor nodes due to belated charging is the first task in this situation.

Since the MCD has enough energy, the main factors influencing the sleep time of the sensor node are the charging sequence and the remaining energy of the sensor node. To minimize the sleep time of the sensor node, an algorithm is proposed to arrange the charging order by considering the position and the remaining energy of the sensor node in the set $V_{\mathrm{minreq}}$.

After the MCD receives the position information of each sensor node, it will calculate the distance between itself and the sensor node. The MCD maintains two separate priority queues: energy priority and distance priority. According to the remaining energy of the sensor node and its relative distance from the MCD, the MCD calculates energy priority $p_{e}\left(i\right)$ and distance priority $p_{d}\left(i\right)$ of sensor node $s_{i}$. By considering these two factors, the priority p(i) of the sensor node $s_{i}$ is:

(18) $$p\left(i\right)=\beta p_{e}\left(i\right)+\left(1-\beta \right)p_{d}\left(i\right),$$

where 0<β<1 represents the weight of the energy priority while (1−β) represents the weight of the distance priority. According to different environments of wireless sensor network [29], through adjusting the value of β and charging the sensor node with the highest priority, different charging targets can be achieves. The charging policy is given in Algorithm 3.

As $B_{\max}>E_{mr}$, after charging sensor nodes in $V_{\mathrm{minreq}}$, the MCD still have some energy for other sensor nodes who also send the charging request. Referring to some existing work, when the energy is uniformly distributed over the whole sensor network, the network can achieve a better network lifetime. For further work of the network, we use a partial charging policy given in Algorithm 2 to plan charging work for the sensor node out of the set $V_{\mathrm{minreq}}$.
**Algorithm 3** Algorithms to minimize the dead time1:**Input**:$V_{\mathrm{minreq}}$, $V_{out}$ and $E_{mr}$.2:**Output**: Charging sequence and total death time.3:Calculate the charging set $V_{\mathrm{charge}}=V_{\mathrm{minreq}}+V_{out}$;4:If $s_{i}\in V_{minreq}$, then5:For $i=1:\left|V_{minreq}\right|$6:Calculate the charging priority of sensor node *i* in $V_{\mathrm{minreq}}$ according to Equation (18);7:End for.8:Sensor nodes in $V_{\mathrm{minreq}}$ are sorted in descending order of $p_{i}$;9:Sensor nodes are charged according to the new order and record the sensor node death time.10:Else Algorithm 2 is used to charge sensor nodes in set $V_{out}$;11:For $i=1:\left|V_{out}\right|$12:Record the sensor node death time;13:End for.14:Update the charging set $V_{\mathrm{charge}}$;15:Calculate the total death time of the sensor node in set $V_{\mathrm{charge}}$.

### 5.4. The Algorithm Maximizing Charging Efficiency

The charging sequence is determined by the remaining energy and position of the sensor node while the charging amount of each sensor node is determined by the remaining energy, energy consumption rate, and the remaining energy of the MCD. Under the premise of maintaining the coverage of target nodes in the network, more sensor nodes are maintained, and then the network performance can be further improved. Hence, to keep more sensor nodes alive, if the MCD has extra energy for sensor nodes outside the set, partial charging is used for these outside sensor nodes.

In Case 3, the MCD has enough energy to serve the entire network, thus the goal of the charging strategy for this case is to maximize charging efficiency on the premise of ensuring the lifetime of the network. The network is considered viable when all target points are covered. The sensor nodes in $V_{\mathrm{minreq}}$ are charged preferentially to ensure that the network functions are complete. To guarantee the normal work of the network, Algorithm 2 is used by the sensor node in $V_{\mathrm{minreq}}$ and the MCD. Then, the outside sensor nodes are charged. Supplementing the energy of sensor nodes outside the set can maintain more sensor nodes for overlay target awareness and further improve network performance. In order to ensure that the same remaining MCD energy can be used to supplement more sensor nodes, Algorithm 4 is proposed to determine the charging sequence and energy proportion for other sensor nodes of the network.

According to Equations (11) and (12), charging efficiency of the MCD is expressed as:

(19) $$\eta =\frac{E_{r,MCD}-\sum_{l=0}^{L-1}e_{0}d_{l,l+1}}{E_{r,MCD}}.$$

In a fixed time, less energy consumed during the MCD charging journey means more energy used to charge the sensor nodes, and the higher energy efficiency of the MCD. That is, the charging utility of the MCD is also related to the travel distance. To maximize the charging efficiency of the MCD, Algorithm 4 considers the residual energy and the energy consumption rate of the sensor node and the position of both sensor node and the MCD to arrange the charging order and determine the charged energy of the sensor node.
**Algorithm 4** Algorithms to maximize charging efficiency1:**Input**: Q, $V_{\mathrm{minreq}}$, $B_{\max}$, $C_{i}$, and $E_{i,t}$.2:**Output**: Charging node sequence and the travel distance.3:For $i=1:\left|V_{minreq}\right|$4:Calculate the remaining energy of MCD $E_{MCD}^{r}=B_{\max}-E_{r,MCD}^{V_{\min req}}$;5:End for.6:Find the set of sensor nodes outside the minimum energy demand node set $V_{res}=V-V_{minreq}$;7:For $i=1:\left|V_{res}\right|$8:Calculate the charging amount of the sensor node to be charged in $V_{res}$ according to Equation (17).9:Calculate the charging efficiency gain of each sensor node MCD.10:End for.11:The final charge efficiency gain $\delta_{i}$ is determined by maximizing the total charge efficiency gain.12:Update the charging set $V_{\mathrm{res}}$;13:For $i=1:\left|V_{res}\right|$14:Calculate $\delta_{i}$ by using $E_{i,ch\arg e}$;15:End for.16:The sensor nodes are sorted according to the charging efficiency gain $\delta_{i}$;17:Derive the driving path of the MCD and the energy of the sensor node $s_{i}$ to be charged.

## 6. Performance Evaluation

In this section, we use MATLAB to verify the proposed algorithm and discuss the effectiveness of the proposed algorithm from different perspectives, including network size, charging rate, maximum data rate, and the number of target monitoring sensor nodes. The results show that the proposed algorithm can effectively improve the network lifetime and the energy efficiency of the MCD.

### 6.1. Parameter Setting

We refer to the parameter setting of the existing work, and deploy the network in the area of 1000 m × 1000 m [19]. The battery capacity of each sensor node is 10.8 KJ [30], and the data rate of the sensor node varies within the interval [1 kbps, 10 kbps] [30]. The number of target nodes in the network is 10. When the target node is not covered by the sensor node, the network is dead. The vehicle speed is 5 m/s [31] and the energy consumption of the vehicle is 0.6 KJ/m [32]. Though the charging rate for some device is small, such as Bluetooth [33], for the approximate charging time and considering charging loss, the transmit power of the MCD is larger and set as any integer within the range [1 Watt, 10 Watts] referring to [3]. The network charging threshold is 5 h, and the charging cycle is one year [34]. In the particle swarm optimization algorithm, the particle size is set to 50, and the maximum number of iterations is set to 1000. Table 2 lists the setting of parameters. To verify the performance, we compared the proposed algorithm with two other algorithms: CSER [19] and JA [35].

### 6.2. Results

To investigate the impact of network configuration on the network lifetime and the charging efficiency, we investigated the impact of different factors on the performance of the proposed algorithm, including network size, charging rate, maximum data rate, and number of target monitoring sensor nodes. In these figures, PA is our proposed scheme.

**Figure 5:**
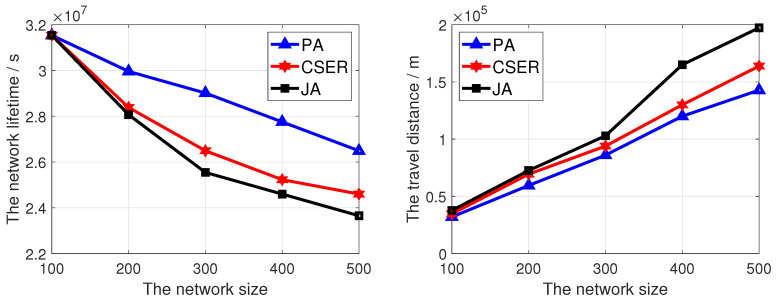
The network lifetime and travel distance of the MCD of proposed algorithm under different network sizes.

Firstly, we simulated the network lifetime and the travel distance under different numbers of sensor nodes: 100–500, and the results are given in Figure 5. Among them, $P_{out}$ is 5 Watts and the maximum data rate is 10 kbps. In Figure 5, with the increase of the network scale, the network survival time gradually decreases, because with the increase of the number of sensor nodes in the network, the number of sensor nodes which need to be charged also increases. At this point, the MCD cannot charge sensor nodes in time, resulting in the death of some sensor nodes used to cover target nodes. At the same time, it can be found that, compared with the other two algorithms our proposed scheme has the maximum network lifetime. This is because the network lifetime is determined by the time the target node is covered, and the algorithm proposed in this paper assigns a higher weight to the sensor node responsible for covering the target node in the charging process. At the same time, the proposed scheme considers that the energy of the MCD may not meet the minimum energy demand in a single charging cycle, and adopts partial charging for the sensor nodes to maintain the maximum possible network operation. Therefore, the network lives longest.

In Figure 5, it can be seen that the travel distance increases with the increase of network size. This is because the number of sensor nodes to be charged increases with the increase of network size, and the travel distance increases. At the same time, the proposed algorithm has the shortest driving distance, because in some cases, such as $E_{mr}>B_{\max}$, compared with other algorithms, partial charging is preferred to meet the sensor nodes within the minimum energy demand set covering the target node for charging. Furthermore, the remaining energy and charging distance of the sensor nodes to be charged are also taken into account, so the charging distance is the shortest. Thus, the moving time of the MCD decreases, which ensures the survival of the network as far as possible.

**Figure 6:**
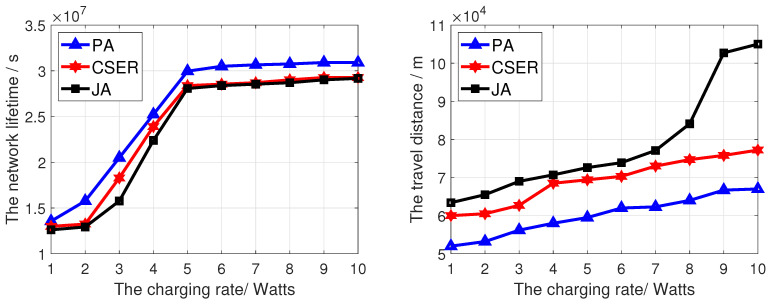
The network lifetime and travel distance of the MCD of proposed algorithm under different charging rate.

Figure 6 analyzes the influence of MCD charging rate on network lifetime and the MCD travel distance. At this point, we set the network size to 200 and the maximum data rate to 10 kbps. In Figure 6, the network lifetime prolongs with the increase of charging rate. This is because the higher the MCD charging rate, the faster the charging speed, and the MCD can replenish energy for the remaining sensor nodes to be charged faster, thus reducing the probability of sensor node death. At the same time, it can be seen that the network lifetime obtained by our algorithm is the highest, because compared with other algorithms, we give priority to the sensor node covering the target node. In addition, partial charging of node fairness is adopted when demand exceeds supply, so as to ensure the coverage of target nodes and prolong the network lifetime. In Figure 6, since this paper comprehensively considers the charging priority of sensor nodes covering target nodes, the remaining energy of sensor nodes to be charged, and the charging distance, the MCD charging distance obtained by the proposed algorithm is the shortest.

**Figure 7:**
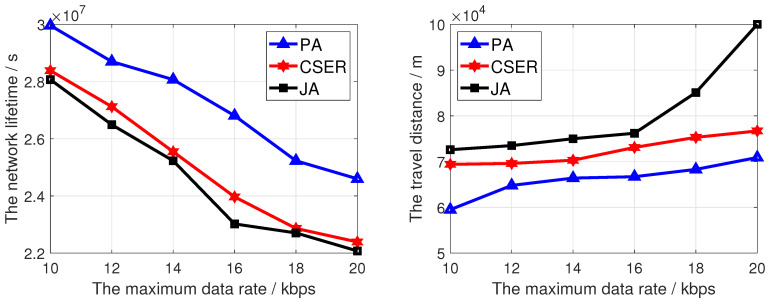
The network lifetime and travel distance of the MCD of proposed algorithm under different maximum data rate.

Figure 7 analyzes the influence of the maximum data rate on the network lifetime and the travel distance, in which the network size is 200 and the charging rate is 5 m/s. In Figure 7, as the data rate increases, the network lifetime decreases, because as the maximum data perception rate increases, the sensor nodes perceive more data in the same time, and the network consumes more energy, thus reducing the network lifetime. At the same time, it can be seen intuitively that the proposed algorithm obtains the highest network lifetime. In Figure 7, as the data rate increases, the travel distance increases. This is because the perceived data quantity of the sensor node increases and the number of sensor nodes to be charged also increases. Therefore, in the same charging cycle, the travel distance of the MCD increases with the increase of the data rate. Furthermore, as this paper comprehensively considers the coverage of target monitoring nodes, residual energy and charging distance, the travel distance is the shortest.

**Figure 8:**
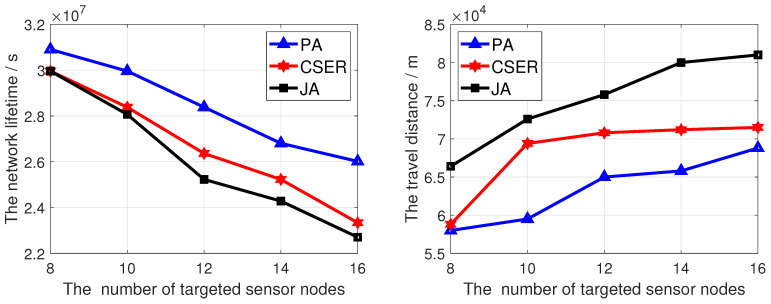
The network lifetime and travel distance of the MCD of proposed algorithm under different number of targeted sensor nodes.

Figure 8 analyzes the influence of the number of target points in the network on the network lifetime and the travel distance. In this figure, the network size is 200, the charging rate is 5 m/s, and the maximum data rate is 10 kbps. In Figure 8, with the increase of the number of target points, the network survival time decreases, because with the increase targets, the probability of the target node failing to be covered the same increases, thus reducing the network lifetime. At the same time, it can be seen that, since this paper considers the charging priority of the sensor nodes to be charged covering the target nodes, and adopts the fair partial charging strategy based on the comprehensive decision of sensor node remaining power, sensor node energy consumption rate, and the MCD remaining energy, the proposed algorithm obtains the highest network lifetime. In Figure 8, as the number of targets increases, the travel distance also increases. This is because as the number of target monitoring nodes increases, the number of sensor nodes to be charged covering the number of target nodes also increases, so that the travel distance increases in the same charging cycle. At the same time, as this paper comprehensively considers the coverage of target nodes, residual energy, and charging distance, the travel distance is the shortest.

**Figure 9:**
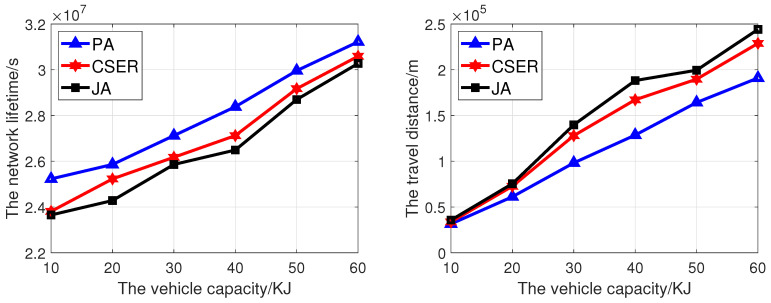
The network lifetime and travel distance of the MCD of proposed algorithm under different MCD capacities.

Figure 9 analyzes the influence of the MCD capacity on the network lifetime and the MCD traveling distance. The network size is 200, the charging rate is 5 m/s, and the maximum data rate is 10 kbps. In Figure 9, as the MCD capacity increases, the network lifetime also increases. This is because as the MCD capacity increases, the number of sensor nodes that can be charged within a cycle increases, more sensor nodes can get energy supplement, and the probability of node death is reduced. Compared with other algorithms, the proposed algorithm has the highest network lifetime, because it gives priority to the minimum energy demand set and designs the corresponding algorithm according to the relationship between supply and demand, effectively prolonging the network lifetime. In Figure 9, as the capacity of the MCD increases, the driving distance of the MCD increases. However, as the proposed algorithm comprehensively considers the set of nodes with minimum energy demand, the coverage of target nodes, remaining power, and charging distance, MCD has the shortest driving distance.

**Figure 10:**
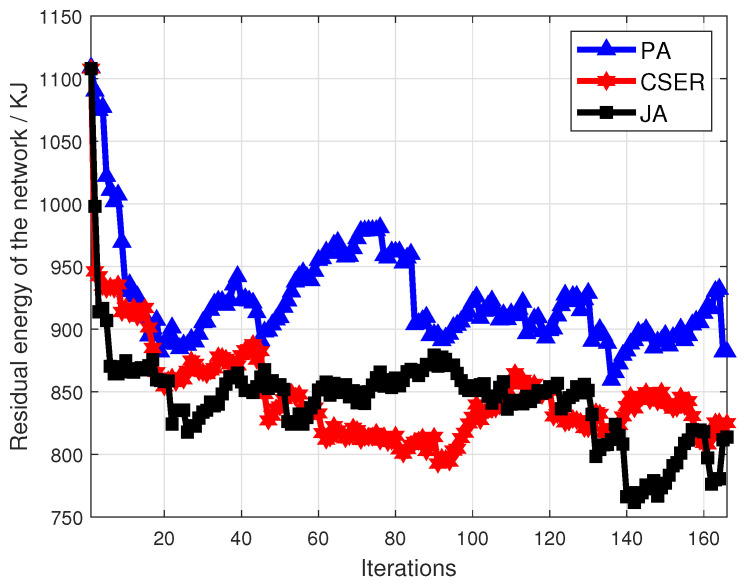
Remaining energy of the network.

Finally, the remaining energy of the network after different iterations is observed, as shown in Figure 10. Compared with CSER and JA, the algorithm proposed in this work has a better effect in preserving network residual energy. This is because PA gives priority to energy supplement for the sensor nodes that cover the target nodes, and also designs fair partial charging during the short supply season. The MCD can charge more sensor nodes, thus effectively prolonging the life time of the network, and the performance of the remaining energy of the network is better under different iterations.

## 7. Conclusions

In this paper, we design a novel charging scheduling scheme based on an energy-constrained MCD to maximize network lifetime. Different from charging directly to all sensor nodes, we first try to use the minimum energy cost to guarantee the sustainable work of the network, and then the remaining energy is used to further improve network performance, such as energy efficiency. To find this minimum cost, an algorithm is proposed to find the minimum sensor nodes set with minimum energy requirement based on particle swarm optimization algorithm. According to the relation among the energy capacity of the MCD, the minimum energy requirement, and the energy requirement of all sensor nodes, the charging problem is divided into three cases. In different cases, maximizing network lifetime is changed into different problems. To solve these problems, three algorithms are proposed to realize the target of maximizing network lifetime and charging efficiency. Though we have considered the lowest cost problem and the role which sensor nodes play in the network in charging strategy, the joint node scheduling and charging should be further carefully designed based on this work in the future.

## Figures and Tables

**Table 1:** Meanings of symbols.

Symbols	Meanings
$L_{1}\times L_{2}$	Size of the WRSN
*N*	Number of sensor nodes
*M*	Number of target points
$S=\left\{s_{1},s_{2},\ldots ,s_{N}\right\}$	Set of sensor nodes
$P=\left\{p_{1},p_{2},\ldots p_{M}\right\}$	Set of target points
$R_{s}$	Sensing radius
$R_{c}$	Communication radius
$B_{ij}$	Coverage relation between target point $p_{j}$ and sensor node $s_{i}$
$d_{ij}$	Distance between target point $p_{j}$ and sensor node $s_{i}$
$\lambda_{i}$	Data generation rate of sensor node $s_{i}$
$E_{\max}$	Energy capacity of the sensor node
$B_{\max}$	Energy capacity of the MCD
$V_{M}$	Moving speed of the MCD
$e_{s}$	Energy consumed by sensing one bit data
$e_{r}\left(d_{ji}\right)$	Energy consumption of receiving the unit data from sensor node $s_{j}$ to sensor node $s_{i}$
$e_{t}\left(d\right)$	Energy consumption of transmitting the unit data when the distance is *d*
$E_{i,t}$	Residual energy of sensor node $s_{i}$ at the moment *t*
$P_{out}$	The charging power
$V_{\mathrm{minreq}}$	Node set with minimum energy request
$E_{mr}$	The minimum required energy

**Table 2:** Experimental parameters setting.

Parameters	Value
L×L	1000 × 1000 [19]
$E_{\max}$	10.8 KJ [30]
$\lambda_{i}$	[1 kbps, 10 kbps] [30]
$V_{M}$	5 m/s [31]
μ	0.5 [31]
$P_{out}$	[1 Watt, 10 Watts] [32]
$e_{0}$	0.6 KJ/m [32]
$B_{\max}$	15 × 10.8 KJ
Minimal lifetime	5 h

## Data Availability

Data is unavailable due to privacy.
